# 

**Published:** 1891-11

**Authors:** 


### CONTENTS FOR NOVEMBER, 1891.

ORIGINAL COMMUNICATIONS.

The General and Special Medical Societies of America. By ADAM H. WRIGHT, M. D........ 193
Shall we Use the Uterine Sound to Correct Backward Displacements of the Uterus? By CLINTON CUSHING, M. D........ 201

SOCIETY PROCEEDINGS.

Philadelphia County Medical Society........ 204
Philadelphia Polyclinic Medical Society........ 207
American Orthopedic Association........ 220
Buffalo Medical and Surgical Association........ 228

SELECTIONS.

Midwifery and Diseases of Women........ 230
Subcutaneous Injections of Iron........ 232

THERAPEUTIC NOTES.

Metric Formulæ........ 233

EDITORIAL.

The Pan-American Medical Congress........ 235

The Mississippi Valley Medical Association........ 236

PERSONAL.

Dr. E. Arnold Praeger—Dr. and Mrs. Henry Gibbons, Jr.—Dr. M. B. Ward—Dr. John A. Ouchterlony—Dr. Edward Clark........ 238

OBITUARY.

Brigadier-General Joseph B. Brown, M. D., U. S. Army........ 239

JOURNALISTIC NOTES.

*Journal of Balneology and Dietary*........ 239
October Number of the *Alienist and Neurologist*........ 239
*The Daily Medical News*........ 240

MEDICAL COLLEGE NOTES.

The College of Physicians and Surgeons........ 240

SOCIETY MEETINGS.

The Southern Surgical and Gynecological Association........ 241

BOOK REVIEWS.

Transactions of the Medical Society of the State of New York for the Year 1891........ 241
Index Catalogue of the Library of the Surgeon-General's Office, United States Army........ 242
A Text-Book of Bacteriology........ 243
Surgical Bacteriology........ 244
Text-Book of Medical Jurisprudence and Toxicology........ 245
Transactions of the Southern Surgical and Gynecological Association........ 246
Minor Surgery and Bandaging........ 247
Lectures on Tumors from a Clinical Standpoint........ 248
Heredity, Health and Personal Beauty........ 248
The Mother's Hand-Book........ 249
An Elementary Hand-Book on Potable Water........ 250
Tables for Doctor and Druggist........ 250
Uses, Tests for Purity, and Preparation of Chemical Re-agents........ 251
Books and Pamphlets Received........ 251

